# Modelling and observing the role of wind in *Anopheles* population dynamics around a reservoir

**DOI:** 10.1186/s12936-018-2197-5

**Published:** 2018-01-25

**Authors:** Noriko Endo, Elfatih A. B. Eltahir

**Affiliations:** Ralph M. Parsons Laboratory, Department of Civil and Environmental Engineering, Massachusetts Institute of Technology, 15 Vassar Street, Cambridge, USA

**Keywords:** Malaria transmission, Water-resource reservoirs, Environmental conditions

## Abstract

**Background:**

Wind conditions, as well as other environmental conditions, are likely to influence malaria transmission through the behaviours of *Anopheles* mosquitoes, especially around water-resource reservoirs. Wind-induced waves in a reservoir impose mortality on aquatic-stage mosquitoes. Mosquitoes’ host-seeking activity is also influenced by wind through dispersion of $CO_2$. However, no malaria transmission model exists to date that simulated those impacts of wind mechanistically.

**Methods:**

A modelling framework for simulating the three important effects of wind on the behaviours of mosquito is developed: attraction of adult mosquitoes through dispersion of $CO_2$ ($CO_2$ *attraction*), advection of adult mosquitoes (*advection*), and aquatic-stage mortality due to wind-induced surface waves (*waves*). The framework was incorporated in a mechanistic malaria transmission simulator, HYDREMATS. The performance of the extended simulator was compared with the observed population dynamics of the *Anopheles* mosquitoes at a village adjacent to the Koka Reservoir in Ethiopia.

**Results:**

The observed population dynamics of the *Anopheles* mosquitoes were reproduced with some reasonable accuracy in HYDREMATS that includes the representation of the wind effects. HYDREMATS without the wind model failed to do so. Offshore wind explained the increase in *Anopheles* population that cannot be expected from other environmental conditions alone.

**Conclusions:**

Around large water bodies such as reservoirs, the role of wind in the dynamics of *Anopheles* population, hence in malaria transmission, can be significant. Modelling the impacts of wind on the behaviours of *Anopheles* mosquitoes aids in reproducing the seasonality of malaria transmission and in estimation of the risk of malaria around reservoirs.

## Background

Malaria transmission is an intricate function of environment. Alternation in environment may exacerbate malaria risks, with global warming being an example [1–4], and the construction of dam-related reservoirs and irrigated fields being another [5–13]. Understanding the environmental determinants of malaria transmission helps in predicting the seasonality and the future risks of transmission, and hence in designing efficient control programs.

Wind conditions, as well as many other environmental conditions, are likely to influence malaria transmission through the behaviours of *Anopheles* mosquitoes—the vectors of malaria. The responses of adult mosquitoes to wind have been poorly understood. Controversy over field observations exists regarding mosquitoes’ flight responses; some suggest mosquitoes fly upwind [14, 15], and others downwind [16, 17]. Results from laboratory experiments support upwind flights, especially in the presence of odour and heat, but also in the absence of them [18–20]. Within some tens of meters from human bait, mosquitoes are generally believed to fly upwind guided by $CO_2$ plume originating from the humans (upwind flight leads mosquitoes towards higher concentration of $CO_2$) [21–24]. Downwind flight behavior may be prominent for long-distance migration [16, 25].

The influence of wind on the population of *Anopheles* mosquitoes may be especially significant around reservoirs [26]. Aquatic-stage mosquitoes that breed at reservoir shorelines face additional mortality through surface waves in reservoirs. Like turbulence during rainstorms, high waves at a reservoir shoreline provide an unfavourable condition for aquatic-stage mosquitoes [5, 27, 28]. Because surface waves become higher in larger and deeper bodies, the mortality from waves is often unique to large water bodies such as reservoirs, but not to small rain-fed puddles.

This paper aims (1) to model the role of wind in the behaviours of *Anopheles* mosquitoes based on physics and physiology known to date, and (2) to quantify the role of wind using observations. To the best of the authors’ knowledge, no mechanistic model exists that incorporates the effect of wind on malaria transmission, except for site-to-site deductive correlation-based models. The *Anopheles* population data come from a village adjacent to the Koka Reservoir in Ethiopia [26]. The impacts of wind on mosquitoes behaviours are incorporated in a malaria transmission model, HYDREMATS—one of the most detailed mechanistic malaria models to date [29]. The role of wind in *Anopheles* population was analysed combining simulations and observations.

## Methods

### Field observations

Multi-year extensive field surveys were conducted near a village adjacent to Koka Reservoir (N8° 25′; E39° 05′) in Ethiopia. The village, named Ejersa, is located north-west of the Koka Reservoir. Its elevation is around 1600 m, and its mean annual temperature is about 21.1 °C. The annual malaria incidence rate in this village was 55 [cases/1000 persons/year] between 2009 and 2014 (personal communication). Of the cases, approximately two-thirds are caused by *Plasmodium falciparum* (*P. falciparum*) and one third by *Plasmodium vivax* (*P. vivax*). The major *Anopheles* (*An.*) vectors present at Ejersa are *An. arabiensis*, *An. pharoensis*, *An. funestus*, and *An. coustani* [9, 26]. Malaria in this area is classified as hypo-endemic, and the incidence rate is declining owing to control measures in place (free diagnosis and medicines, distribution of insecticide-treated bed nets, occasional use of indoor residual spraying).

**Fig. 1:**
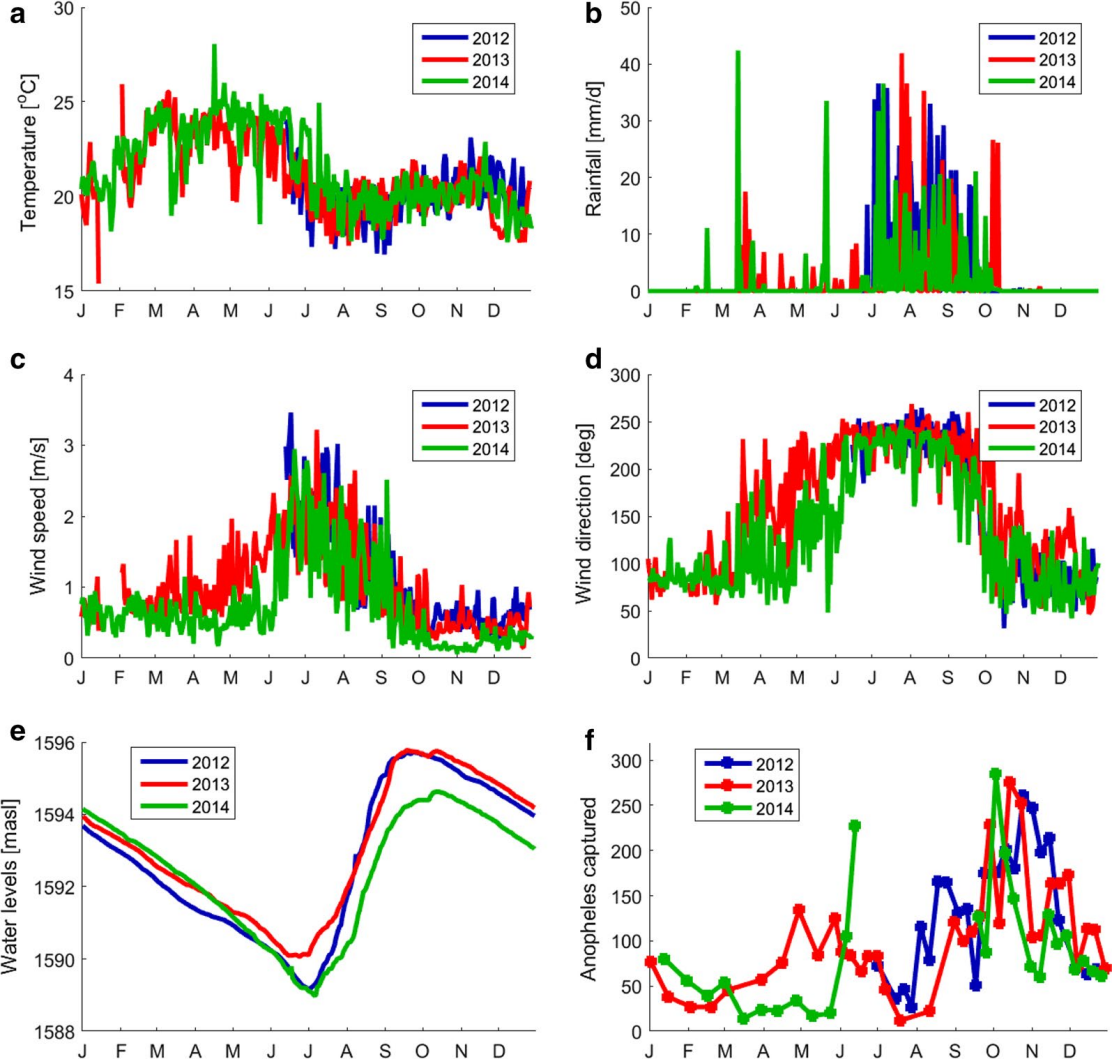
Observed environmental and entomological conditions in Ejersa. Temperature (**a**), rainfall (**b**), wind speed (**c**), and wind direction (**d**) were observed at an in situ weather station at 30-min resolution, and the daily-average data are presented here. Wind direction was measured with respect to the north, increasing clockwise. The operation of the weather station started in Jul. 2012. The daily reservoir water levels were obtained from EEPCo and presented in meters above sea level [masl] (**e**). Observed *Anopheles* population in the six light traps are shown in square, and the data points are connected with simple linear interpolation (**f**). Observational *Anopheles* population data are not available during Jan.–June 2012 and Jul.–Aug. 2014. Through **a**–**f** the data in 2012, 2013, and 2014 are presented blue, red, and gree, respectively

The field campaigns span from Jul. 2012 to Apr. 2015, monitoring environmental and entomological conditions [26]. Detailed information on local wind profile (wind speed and wind direction) were obtained from an *in situ* weather station at 30-min resolution, as well as other climatological data (Fig. 1a–d). The daily water levels of the Koka Reservoir were obtained from the Ethiopian Electric Power Corporation (EEPCo) (Fig. 1e). *Anopheles* population dynamics were monitored through weekly or bi-weekly adult sampling surveys using six CDC miniature light traps deployed in Ejersa (Fig. 1f).

This area experiences three climatological seasons: a main rainy season from June to September, locally known as *Kiremt*; a dry season from October to February, *Bega*; and a secondary rainy season from March to May, *Belg* (Fig. 1b). During the main rainy season, the temperature becomes lower than the other two seasons (Fig. 1a). Wind profile also shifts between the rainy seasons and the dry season (Fig. 1c, d). The reservoir water levels have the lowest and highest peaks around the beginning and the end of the main rainy seasons (Fig. 1e). The *Anopheles* population peaks once or twice a year (Fig. 1f). A large increase in population occurs during Sep.–Dec. (hereafter the *major mosquito season*). A small increase may or may not occur during May.–June (hereafter the *minor mosquito season*). How the *Anopheles* population is influenced by the environmental conditions in Ejersa is described in Endo and Eltahir [26].

### Modelling mosquitoes’ flight behaviours

The locomotion of adult mosquitoes ($\overrightarrow{v}$) is modelled as a combination of active dispersal ($\overrightarrow{v_{active}}$) and passive dispersal ($\overrightarrow{v_{passive}}$). Active dispersal ($\overrightarrow{v_{active}}$) is also called appetential dispersal, where mosquitoes fly around by themselves in search of blood sources and oviposition sites, for example. Passive dispersal ($\overrightarrow{v_{passive}}$), on the other hand is called non-appetential dispersal, where mosquitoes move with wind through advection.

1 $$\begin{aligned} \overrightarrow{v}=\overrightarrow{v_{active}}+\overrightarrow{v_{passive}} \end{aligned}$$

### Modelling the role of wind

Three important effects of wind on the behaviours of mosquitoes are modelled: attraction of adult mosquitoes through dispersion of $CO_2$ (hereafter, $CO_2$ *attraction*), advection of adult mosquitoes (hereafter, *advection*), and aquatic-stage mortality due to wind-induced surface waves (hereafter, *waves*).

#### $CO_2$ attraction

Mosquitoes active flight component ($\overrightarrow{v_{active}}$) can be modelled as a summation of random flight ($\overrightarrow{v_{random}}$) and directed flight ($\overrightarrow{v_{directed}}$).

2 $$\begin{aligned} \overrightarrow{v_{active}}=\overrightarrow{v_{random}}+\overrightarrow{v_{directed}} \end{aligned}$$

Mosquitoes use various olfactory sensors, visual sensors, and thermal sensors to identify the locations of hosts; the signal that travels the farthest is $CO_2$. A $CO_2$ plume moves downwind with turbulent diffusion [22, 30]. When mosquitoes sense elevated $CO_2$ concentration (activation), they become activated and generally fly upwind, towards the source of $CO_2$ [18–24]. In the absence of the clues of hosts, mosquitoes fly randomly.

The relative importance of $\overrightarrow{v_{random}}$ and $\overrightarrow{v_{directed}}$ is determined by the $CO_2$ concentration and the concentration gradient. Studies show that mosquitoes can sense the fluctuation of $CO_2$ concentration by as little as 40 ppm [31] or 100 ppm [20]. In the experiment by Healy and Copland [20], approximately 60% of mosquitoes were activated and flew upwind when they encountered pulses of 100 ppm or more $CO_2$ above the background level (350–370 ppm). It can be assumed that mosquitoes’ active dispersal is fully random, unless mosquitoes sense at least 40 ppm higher $CO_2$ concentration than the background; with the concentration difference of 40 ppm, 60% of their flight is directed towards the higher concentration of $CO_2$, while the other 40% remaining as the random-direction flight. The weight of the directed flight component was assumed to increase linearly with the concentration gradient, such that mosquitoes located 10 m downwind from a source of $CO_2$ fly directly to the source (i.e., the directed component is 100%), assuming a typical house in Ejersa with five inhabitants and one cow. Using the weight of the directed flight component ($a,~ 0\le a\le 1$) and the average flight velocity of mosquitoes *v*, the magnitude of $\overrightarrow{v_{random}}$ and $\overrightarrow{v_{directed}}$ are modelled as:

3 $$\begin{aligned} |\overrightarrow{v_{random}}|=(1-a)v \end{aligned}$$

4 $$\begin{aligned} |\overrightarrow{v_{directed}}|=av. \end{aligned}$$

The direction of $\overrightarrow{v_{directed}}$ is toward the steepest $CO_2$ concentration gradient, and that of $\overrightarrow{v_{random}}$ is random.

The concentration of $CO_2$ is simulated for time-averaged mean values using the Gaussian dispersion equation [32]:

5 $$\begin{aligned} c(x,y,z) =& \frac{Q}{{2\pi \sigma _{y} \sigma _{z} u}}exp\left( {\frac{{ - y^{2} }}{{2\sigma _{y}^{2} }}} \right) \\ &{\left( {exp\left( {\frac{{ - (z - h)^{2} }}{{2\sigma _{z}^{2} }}} \right) + exp\left( {\frac{{ - (z + h)^{2} }}{{2\sigma _{z}^{2} }}} \right)} \right)}, \\ \end{aligned}$$

where: *c* is the concentration of $CO_2$ [g m^−3^] at any position *x* meters downwind of the source, *y* meters crosswind of the source, and *z* meters above the ground level, *Q* is the carbon dioxide exhalation rate [g s^−1^], *u* is the horizontal wind velocity along the plume centerline [g s^−1^], *h* is the height of the emission plume centerline above the ground [m], $\sigma _z$ is the vertical standard deviation of the emission distribution [m], and $\sigma _y$ is the horizontal standard deviation of the emission distribution [m].

The horizontal and vertical dispersion is a function of atmospheric stability conditions and the downwind distance (*x*) [30]. During the nighttime periods, when mosquitoes are active, atmospheric conditions are stable due to radiative cooling at the land surface under clear skies. From Smith [30], the horizontal and vertical dispersions for stable conditions are given by:

6 $$\begin{aligned} \sigma _y=0.24x^{0.71} \end{aligned}$$

7 $$\begin{aligned} \sigma _z=0.06x^{0.71}. \end{aligned}$$

The height of the emission plume (*h*) was set at 1 m, roughly the level of beds, and the height at which mosquitoes sense the plume (*z*) at the same height (1 m). The source emission of $CO_2$ exhaled is set at 275 ml min^−1^ per human and 3925 ml min^−1^ per cow [33]. Based on field surveys, it is assumed every household compound contains five humans and one cow. The concentration of $CO_2$ at each time step in the model domain is calculated as the sum of the contributions of all exhaling members of the community.

The Gaussian model is a well-established time-averaged model of plume dispersion; however, mosquitoes are known to respond to the instantaneous high concentration of $CO_2$ that is maintained in a pocket of air due to turbulence, rather than the mean concentrations of $CO_2$ [21, 22]. Because of turbulence, a $CO_2$ plume is unevenly distributed in the air with some small eddies containing high concentrations of $CO_2$. Studies with high-resolution measurements observed many-fold higher than mean concentration of $CO_2$ at a frequency of 0.1 to a few seconds [21, 22]. Simulating this small-scale structure of $CO_2$ plumes is computationally expensive. Instead, it is assumed that there exist pockets of $CO_2$ plume with concentration as high as 10 times the concentration simulated in the Gaussian model, and that mosquitoes can respond to the instantaneous burst of $CO_2$.

As the Gaussian dispersion equation demonstrates, the concentration of $CO_2$ depends on the source load of $CO_2$, distance to the source, wind speed, and wind direction. Assuming that *Anopheles* can sense elevated levels of $CO_2$ above 40 ppm (simulated mean concentration of 4 ppm), the maximum range over which *Anopheles* is activated was simulated to be about 100, 50, 30 and 15 m downwind of a house (with five inhabitants and one cow) under 0.5, 1, 2, and 5 m/s of wind, respectively. The effective range of $CO_2$ activation is believed to be within some tens of meters around a host [21, 34–36]. Considering that the ranges simulated are for a house with multiple hosts, simulated ranges agree with literature values.

#### Advection

In addition to mosquitoes’ active flight behaviours, mosquitoes are assumed to move downwind with the wind’s advective effect. This mechanism plays an important role in mosquitoes’ long-distance migration [16, 17]; however, its role is assumed to be negligible if hosts are found near the breeding habitats. In this simulation, mosquitoes were assumed to move downwind, but only at a small fraction (0.01%) of the wind speed:

8 $$\begin{aligned} \overrightarrow{v_{passive}}=0.0001 \overrightarrow {u}, \end{aligned}$$

where $\overrightarrow {u}$ is a wind vector pointing downwind.

**Fig. 2:**
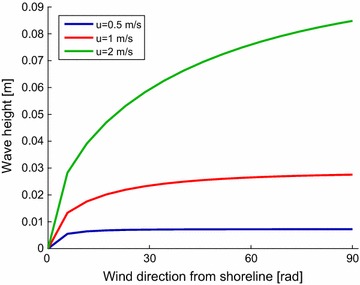
Predicted wave height for Ejersa. The wave heights calculated for the Koka Reservoir using the shallow-water forecasting model are shown as a function of the wind direction with respect to a shoreline. The wind direction determines the fetch. The estimates are made for different wind speeds: 0.5 m/s (blue), 1 m/s (red), and 2 m/s (green). Larger wind speed yields larger waves. The estimates are made using the average water depth of the Koka Reservoir as *d*

#### Waves

High waves exhaust larvae and cause higher mortality [5, 27, 28]. High waves are not likely to occur in small and shallow water bodies, such as rain-fed pools, but could be significant in large and deep water bodies, such as reservoirs. Waves seen at reservoirs are called surface waves and are created by the shear stress generated by wind. The surface waves are larger with higher wind speed, larger fetch, and deeper water. The height of the surface wave ($H_w$ [m]) can be estimated through the shallow-water forecasting model [37] (Fig. 2). The model is based on theoretical assumptions and successive approximations in which wave energy is added due to wind stress and subtracted due to bottom friction and percolation:

9 $$\begin{aligned} \frac{gH_w}{u^2} =0.283 ~ \tanh \bigg ( 0.530 \Big ( \frac{gd}{u^2} \Big )^{3/4} \bigg ) \\~ \tanh \Bigg ( \frac{0.00565 \Big ( \frac{gF}{u^2} \Big )^{1/2}}{\tanh \bigg ( 0.530 \Big (\frac{gd}{u^2} \Big )^{3/4} \bigg )} \Bigg ), \end{aligned}$$

where: *u* is the wind speed [m s^−1^], *d* is the depth of water [m], *F* is the fetch, the length of water over which the wind blows in a single direction [m], and *g* is the gravitational constant [m^3^ kg^−1^ s^−2^].

For simplicity, the average water depth of a reservoir is used as *d*. The fetch, *F*, is calculated assuming that a reservoir is circular.

**Fig. 3:**
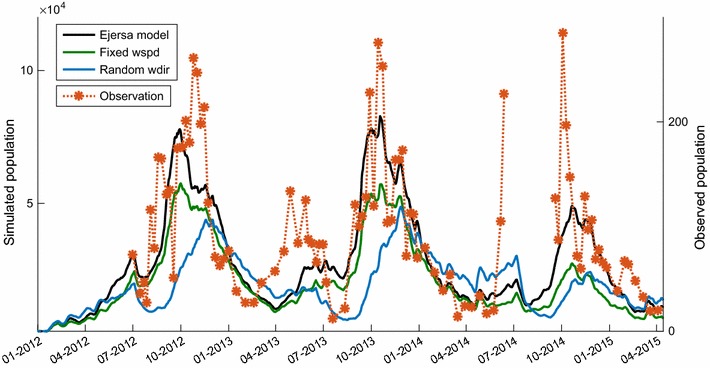
Simulated and observed *Anopheles* population. The total size of the *Anopheles* population simulated in the simulation domain is shown on the left y-axis for the Ejersa model (black), the fixed *wspd* model (green), and random *wdir* model (blue). The observed numbers of *Anopheles* captured in the six light trap houses are shown on the right y-axis; observed values are presented in orange asterisks and orange dotted lines are simple linear interpolation. Observational data are not available during Jan.–June 2012 and Jul.–Aug. 2014

$H_w$ was then converted into mortality ($m_{wave}$ [h^−1^]), assuming the mortality linearly increases with wave height:

10 $$\begin{aligned} m_{wave}=f\times H_w, \end{aligned}$$

where *f* is the conversion factor [h^−1^ m^−1^]. *f* was set at 0.1 as a result of calibration using observational data.

Expected wave height at shorelines of the Koka Reservoir is shown in Fig. 2 for* u* = 0.5 (blue), 1 (red), and 2 m/s (green) and various angles of wind (x-axis). The in situ wind sentry recorded that the daily wind speed in Ejersa varied between 0.5 and 2 m/s in a year. The observed wave heights at the centimetres, which is in good agreement with the predicted results.

### Malaria transmission simulator

The role of wind in shaping the behaviours of *Anopheles* mosquitoes was incorporated in HYDREMATS [26, 32] to test the accuracy of the model, comparing with observations. HYDREMATS is a village-scale malaria transmission model that features explicit representation of evironmental conditions and behaviours of *Anopheles* mosquitoes in space and time. Its agent-based approach is suitable for employing the role of wind described above. HYDREMATS was tailored for Ejersa (hereafter, *Ejersa model*) [26].

In order to examine the role of wind in the dynamics of *Anopheles* population, the Ejersa model was also forced with fixed wind speed (*fixed wspd model*) or with random wind direction (*random wdir model*) instead of respective observational values. In the fixed *wspd* model, the observed mean wind speed (0.884 m/s) was employed for every timestep (1 h) throughout the simulation period. The impact of wind speed and direction can be understood by the deviation between the Ejersa model and the respective simulation.

## Results

### Observation of environment and *Anopheles* population dynamics

Temperature and rainfall are often described as the primary determinants of *Anopheles* population dynamics [3, 38, 39]; however, in Ejersa, reservoir water levels and wind profile are likely to be more important [26] (Fig. 1). Around the temperature range in Ejersa (19–24 °C), the expected longevity of *Anopheles* is not sensitive to temperature [40, 41]. Thus, the influence of temperature on the *Anopheles* population dynamics is limited. Field observations found a handful of rain-fed puddles in Ejersa, but only a few of them were positive breeding sites. This is because rain-fed puddles rarely persist long enough to support the completion of *Anopheles’* aquatic-stage development, which takes around 15–20 days at the cold range of temperature in the field. [26]. The lag in observed *Anopheles* population dynamics was too large to be explained by the rainfall (Fig. 1b, f). On the other hand, the primary determinant of the *Anopheles* population was analysed to be the reservoir water levels (Fig. 1e, f), which determine the location of the reservoir shoreline [26]. The shoreline—the main breeding habitat for *Anopheles* mosquitoes—becomes closer to the village as the reservoir water levels increase, making reproduction more likely.

The observed *Anopheles* population dynamics during the minor mosquito season (around May, more precisely) were distinctive between 2013 and 2014 (Fig. 1f). The mosquito population increased in 2013 but not in 2014. Neither temperature, rainfall, nor reservoir water level is likely to explain the difference, because the observed data during the same season in the two years were similar. The noticeable differences between the two periods were found only in wind speed and wind direction (Fig. 1c, d, respectively). Whether or not this observational differences in wind profile can explain the observed *Anopheles* population dynamics during the minor mosquito season are examined mechanistically in the next section.

### Simulation of *Anopheles* population dynamics

Simulated and observed *Anopheles* population are presented in Fig. 3. The observed dynamics of *Anopheles* population (orange asterisks) were reproduced in the Ejersa model (solid black line), both during the major and minor mosquito seasons, with some reasonable accuracy [26]; however, without the incorporation of wind impacts, models (solid green line and solid blue line) failed to reproduce the observed seasonality of *Anopheles* population. The result from the fixed *wspd* model (solid green line) was somewhat similar to that of the Ejersa model, but the performance of the model declined. In the random *wdir* model (solid blue line), the performance of the model declined even more significantly, and the observed dynamics of the *Anopheles* population was not reproduced with this model. The random *wdir* model failed to simulate the timing of the onset of the major mosquito season. In addition, it failed to simulate the observed difference of the *Anopheles* population dynamics during the minor mosquito season.

**Fig. 4:**
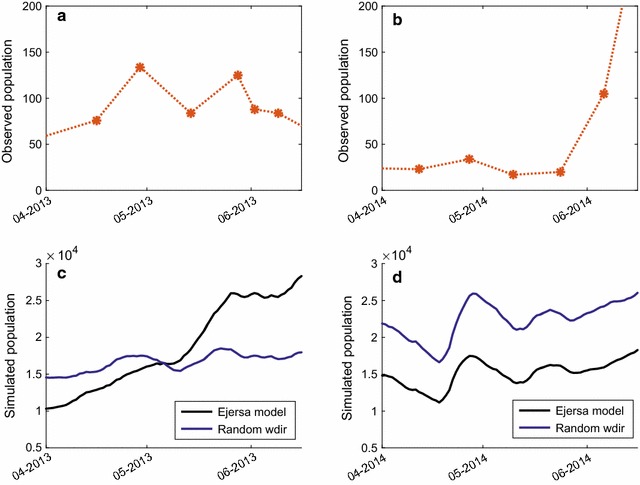
*Anopheles* population in minor mosquito season. Observed (**a**, **b**) and simulated (**c**, **d**) *Anopheles* populations during the minor mosquito season in 2013 (**a**, **c**) and in 2014 (**b**, **d**). An increase in *Anopheles* population was observed in May 2013, but not in May 2014 (**a**, **b**). This difference was simulated with reasonable accuracy in HYDREMATS (**c**, **d**, black line), which can be attributable to different profiles of wind direction. The observed wind direction in 2013 (**c**, black) worked to enhance mosquito population slightly, as compared to an assumed condition with random wind direction (primary determinan, blue). The observed wind direction in 2014, on the other hand, worked to suppress the mosquito population (**d**, black), as compared to an assumed condition with random wind direction (**d**, blue)

The observed *Anopheles* population increased in May in 2013, but not in 2014 (Fig. 4a, b). This difference was simulated in the Ejersa model, and the simulation results suggest that it is accounted for mostly by the wind direction (Fig. 3). Figure 4 demonstrates that the observed wind direction in the minor mosquito seasons in 2013 worked to enhance mosquito population (c, black) slightly, as compared to an assumed condition with random wind direction (c, blue). The observed wind direction in the minor mosquito season in 2014, on the other hand, worked to suppress mosquito population (d, black), as compared to an assumed condition with random wind direction (d, blue).

## Discussion

The role of wind in mosquito behaviours was modelled based on the physics of $CO_2$ dispersion and surface waves and on the physiology of mosquitoes. The mosquitoes’ behavioural responses to wind are still not fully understood; however, the model incorporating some of the known effects of wind speed and wind direction was demonstrated to be able to reproduce the observed dynamics of *Anopheles* population in Ejersa, which was not possible without those wind effects. Thus, the impacts of wind on the mosquitoes’ behavioural responses are believed to be credibly represented in this analysis. Some of the model parameters may still benefit from further calibration. To the best of the authors’ knowledge, HYDREMATS is the only malaria transmission model that mechanistically incorporates the effect of wind.

The effect of wind on the *Anopheles* population dynamics has received limited attention in the scientific community [20, 21, 23, 24], yet it was shown to have a significant contribution around a reservoir. The importance of wind is expected to be particularly significant around reservoirs for two reasons. The first reason is that the waves created by the wind can become fatal to aquatic-stage mosquitoes at large water bodies. The height of the wave increases with the depth and the fetch (~ surface area) of the reservoir. Thus, waves are more likely to influence *Anopheles* mosquitoes’ breeding at reservoirs than at small water bodies such as rain-fed pools. The second reason is the heterogeneity in the surrounding environment around reservoirs, where human settlements are located only at one side of the shoreline. Under such environment, the population dynamics of *Anopheles* mosquitoes are likely to be influenced by the wind direction.

As compared to offshore wind, onshore wind creates larger waves because the fetch is large. Thus, aquatic-stage mosquitoes experience larger mortality, leading to smaller *Anopheles* populations. In addition, under the onshore wind, a large part of $CO_2$ plume emanated from the village moves away from the reservoir, which makes mosquitoes emerging at reservoir shorelines less efficient in identifying the direction of the village for host-seeking. Thus, under onshore wind, *Anopheles* population can further decrease due to limited $CO_2$ attraction and less efficient host-seeking activity. These two factors conclude that *Anopheles* populations are generally low under the onshore wind condition.

Wind direction in Ejersa shifts from about 90° north in the dry season to about 220° north in the main rainy season. A gradual shift of wind direction is experienced during the secondary rainy season. The wind from 90° and 220° north corresponds to onshore wind and near-offshore wind for Ejersa. As a results, part of the increase in the *Anopheles* population during the main rainy season (beginning of the main mosquito season, more specifically) can be explained by the wind blowing from near-offshore (Fig. 3). The difference in the *Anopheles* population dynamics during the minor rainy season (which almost corresponds to the secondary mosquito season) between 2013 and 2014 can also be explained by the wind direction. In 2013, the shift in wind direction during the minor rainy seasons occurred earlier than in 2014 (Fig. 1), explaining the observed and simulated difference in the *Anopheles* population dynamics.

The model that replaced the observed wind speed with the averaged wind speed (fixed wind model) consistently simulated smaller *Anopheles* population than the model with observed wind speed (Ejersa model) throughout a year. This unexpected result can be explained by the fact that *Anopheles* mosquitoes are modelled to be active only during the night time, and the “average” wind speed averages the observed profile not only over the seasons but also over day and night. The day-time wind speed (~ 1.0 m/s) was larger than the night-time wind speed (~ 0.65 m/s). Thus, the average wind speed was larger than the night-time wind speed. Larger wind speed enhances waves and does not deliver high concentration of $CO_2$ plume far enough—both mechanisms contribute to decrease the *Anopheles* population. Thus, the fixed *wspd* model resulted in consistently smaller *Anopheles* population than the Ejersa model throughout the simulation period.

## Conclusion

Around large water bodies such as reservoirs, the role of wind in *Anopheles* population dynamics, hence in malaria transmission, can be significant. This paper provided a framework to model the effects of wind in the behaviours of *Anopheles* mosquitoes. The effects important for *Anopheles* behaviours include: attraction of adult mosquitoes through dispersion of $CO_2$, advection of adult mosquitoes, and aquatic-stage mortality due to wind-induced surface waves. Combining simulation studies and observational data of *Anopheles* population dynamics collected around the Koka Reservoir in Ethiopia, this study demonstrates a substantial role of wind in *Anopheles* population dynamics—hence the dynamics of malaria transmission. It is suggested that malaria is generally suppressed when wind blows from a reservoir to a village.
